# The Role of Gaseous Molecules in Traumatic Brain Injury: An Updated Review

**DOI:** 10.3389/fnins.2018.00392

**Published:** 2018-06-08

**Authors:** Xiaoru Che, Yuanjian Fang, Xiaoli Si, Jianfeng Wang, Xiaoming Hu, Cesar Reis, Sheng Chen

**Affiliations:** ^1^Department of Cardiology, Zhejiang Provincial People’s Hospital, People’s Hospital of Hangzhou Medical College, Hangzhou, China; ^2^Department of Neurosurgery, The Second Affiliated Hospital, School of Medicine, Zhejiang University, Hangzhou, China; ^3^Department of Neurology, The Second Affiliated Hospital, School of Medicine, Zhejiang University, Hangzhou, China; ^4^Department of Neurosurgery, Taizhou Hospital, Wenzhou Medical University, Linhai, China; ^5^Department of Physiology and Pharmacology, Loma Linda University, Loma Linda, CA, United States; ^6^Department of Preventive Medicine, Loma Linda University Medical Center, Loma Linda, CA, United States

**Keywords:** traumatic brain injury, gaseous molecules, neuroprotection, nitric oxide, carbon monoxide, hydrogen sulfide, molecular hydrogen, central nervous system

## Abstract

Traumatic brain injury (TBI) affects millions of people in China each year. TBI has a high mortality and often times a serious prognosis. The causative mechanisms of TBI during development and recovery from an injury remain vague, leaving challenges for the medical community to provide treatment options that improve prognosis and provide an optimal recovery. Biological gaseous molecules including nitric oxide (NO), carbon monoxide (CO), hydrogen sulfide (H_2_S), and molecular hydrogen (H_2_) have been found to play critical roles in physiological and pathological conditions in mammals. Accumulating evidence has found that these gaseous molecules can execute neuroprotection in many central nervous system (CNS) conditions due to their highly permeable properties allowing them to enter the brain. Considering the complicated mechanisms and the serious prognosis of TBI, effective and adequate therapeutic approaches are urgently needed. These four gaseous molecules can be potential attractive therapeutic intervention on TBI. In this review, we will present a comprehensive overview on the role of these four biological gasses in the development of TBI and their potential therapeutic applications.

## Introduction

Traumatic brain injury (TBI) affects 3–4 million people in China every year and accounts for 87% of deaths related to trauma. With its high mortality and serious prognosis, it is the fourth leading cause of death in young people (Liu, 2015). In addition, the incidence of TBI in the elderly appears to be increasing (Peeters et al., 2015). The causative mechanisms of TBI during development and recovery from an injury remain poorly understood. This poses great challenges for medical management following TBI, and leaves the medical community with challenges such as finding new treatment options to improve recovery following TBI (Seule et al., 2015; An et al., 2016).

Currently, various gaseous molecules (such as air content gasses, volatile anesthetics, non-volatile anesthetics, noble gasses) were thought to protect neural system in neurological diseases (Deng et al., 2014). Biological gaseous molecules, also referred as gasotransmitters, including nitric oxide (NO), carbon monoxide (CO), hydrogen sulfide (H_2_S), and molecular hydrogen (H_2_), also serve critical roles in mammals’ physiological and pathological conditions (Zhou et al., 2012). They can easily cross the blood–brain barrier (BBB) and spread through brain tissue due to their smaller molecular weights compared with chemically formulated drugs (Zhou et al., 2012; Deng et al., 2014). Accumulating evidence has demonstrated that these gaseous molecules provide neuroprotection in many diseases of the central nervous system (CNS) through different mechanisms and administration regimens (Ren et al., 2010; Charriaut-Marlangue et al., 2012; Zhan et al., 2012; Otterbein, 2013).

Considering the complicated mechanisms and the serious prognosis of TBI, effective and adequate therapeutic approaches are urgently needed. A better understanding of the physiological function and alterations of gaseous molecules in pathological conditions may provide a potentially attractive therapeutic intervention for TBI. In our review, we will present a comprehensive overview of the role of these four biological gasses in the development of TBI and their potential therapeutic applications.

## Nitric Oxide

Nitric Oxide is the most recognized endogenous gasotransmitter in mammalian biology. It is mainly synthesized during L-arginine conversion with the assistance of three NO synthases (NOS): neuronal (n) NOS, inducible (i) NOS, and endothelial (e) NOS (Forstermann and Sessa, 2012). Despite nNOS being the predominant NO producer in CNS, iNOS, and eNOS can also be expressed in neurons and endothelial cells in the brain (Galea et al., 1992; Olivenza et al., 2000). NOS alteration leads to cerebral NO level changes and was found to be associated with TBI occurrence and secondary damage after TBI (Stover et al., 2014; Villalba et al., 2017). In addition, NO participates in the regulation of many biological process such as neurogenesis, cerebral blood flow (CBF) maintenance, oxidative stress reactions, and neuronal cell death (Uchiyama et al., 2002; Packer et al., 2003; Toda et al., 2009). Whether or not it has a protective or destructive role in the CNS remains controversial (Ockelford et al., 2016).

During pathophysiological processes in TBI, NO homeostasis is mainly mediated by NOS isoform activity (Cherian et al., 2004). eNOS and nNOS are constitutively expressed in the brain via induction of Ca^++^, while iNOS becomes unregulated during brain damage or injury (Bredt and Snyder, 1990). Though the pathogenesis of TBI is driven by complex mechanisms, it was widely accepted that the inflammatory reaction is the main reason a response is elicited to brain injury (Corps et al., 2015). Inflammatory cascades promote expression of constitutive NOS isoforms and up-regulation of iNOS levels after TBI occurs (Olmos and Llado, 2014).

After TBI, the widespread brain injury may induce cell depolarization, such as rising extracellular potassium and intracellular Ca^++^ (Faden et al., 1989; Bezzi et al., 1998; Folkersma et al., 2011). Currently, studies suggest Ca^++^ accumulation can be mediated by the glutamate wave that follows TBI (Kawamata et al., 1992; Chamoun et al., 2010). Following inflammatory stimuli, extracellular glutamate concentrations in the brain tissue are markedly increased (Bezzi et al., 1998; Folkersma et al., 2011). These elevated glutamate levels were reported to be related to Ca^++^ influx and cytotoxicity during TBI (Chamoun et al., 2010). With the assistance of receptors such as NMDA receptors, the increased intracellular Ca^++^ consequently reacts with calmodulin and promotes the expression of constitutive NOS (Southam et al., 1991; Zur Nieden and Deitmer, 2006). This process appears in the early time period (30 min) of TBI sequela and contributes to the first NO peak (Marletta, 1994; Wada et al., 1998a). In addition, the NO produced by different constitutive NOS synthases may each contribute uniquely to the outcome. Activation of nNOS induces toxic effects that produce excitotoxicity and oxygen free radicals, such as Peroxynitrite (ONOO^-^) to cause cell death (Wada et al., 1998a; Gahm et al., 2002). nNOS-derived NO is also involved in synaptic plasticity and neuronal signaling after TBI (Garthwaite, 1991). The function of eNOS-derived NO works against the toxic effect of nNOS-derived NO (Gahm et al., 2002). It participates in cerebrovascular responses by dilating blood vessels to maintain CBF (Goadsby et al., 1992; White et al., 2000). The expression of constitutive NOS isoforms also leads to transient hypertension surge through massive sympathetic discharge (Rosner et al., 1984).

In the later response phase, the inflammatory reaction following TBI induces the expression of iNOS (Minc-Golomb et al., 1994; Heneka and Feinstein, 2001). Normally, the response can be divided into two parts (Cherian et al., 2004). The first iNOS response starts 4 to 6 h after trauma, and peaks 8 to 23 h after TBI (Gahm et al., 2002; Ucal et al., 2017). iNOS expression may be associated with the increased amount of neutrophils and microglia during this response (Royo et al., 1999; Bayir et al., 2005). The second iNOS response starts 72 h after trauma and is related to the immunoreactivity of microglia and macrophages (Orihara et al., 2001). The waves of iNOS response peak 7 days following TBI (Wada et al., 1998b; Jin et al., 2012). The role of iNOS remains controversial, but many studies suggest that the NO released by iNOS can react with superoxide radicals and generate more deleterious reactive species, causing neuronal death and worsening neurological outcome (Sinz et al., 1999; Gorlach et al., 2000; Berka et al., 2014). In contrast to these detrimental effects, iNOS-derived NO also has the ability to attenuate oxidative stress reactions by preventing mitochondrial damage from reactive oxygen species (ROS) and decreasing redox iron activity (Bayir et al., 2005; Dungel et al., 2015).

**FIGURE 1:**
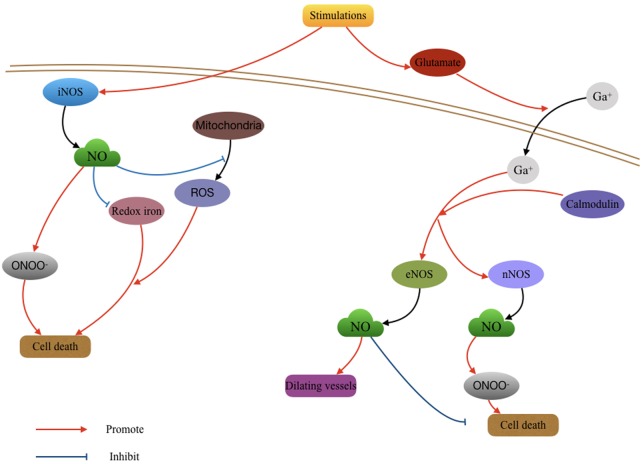
Role of nitric oxide (NO) in the pathological changing after Traumatic brain injury (TBI). The activity of NO produced by different NO synthases (NOSs) function various effects in TBI. The deleterious effects of NO mainly resulted by the oxygen free radicals waves which subsequently caused cell death; the protective effects of NO may include vasodilatation and antioxidant effect.

Currently, the application of inhaled NO in TBI models is being investigated. Using closed head mild TBI mouse models, a study found that mild TBI induced a short-term memory loss and strong inflammatory reaction in the first 24 h after mild TBI. This injury only lasts for 2–3 days. Treatment with a low concentration and short duration (less than 8 h) of inhaled NO could prevent the adverse effects of mild TBI including acute and transient cognitive deficits and inflammation. Whereas, the group treated with a higher concentration of NO for 24 h showed no benefit in memory (Liu et al., 2013). Additionally, inhaled NO was shown to significantly improve CBF and reduce intracranial pressure after TBI in mice. Long duration (24 h) inhalation reduced brain injury and improved neurological function (Terpolilli et al., 2013). Further investigation is warranted for the potential use of inhaled NO after TBI, particularly with regard to dosage and timing of administration (**Figure 1**).

## Carbon Monoxide

Carbon Monoxide, traditionally thought of as a toxic gas, also acts as a gasotransmitter in both the extracellular and intracellular spaces. However, its biological function remains controversial (Coburn et al., 1963; Tenhunen et al., 1968). Heme oxygenase (HO) enzymes, including HO-1 and HO-2 are used in the process of heme degeneration and CO generation (Ewing and Maines, 1993; Li and Clark, 2000). HO-1, also named heat shock protein 32, is an inducible protein upregulated predominantly in numerous conditions of cellular stress. It was found to be up-regulated and play a cytoprotective role against oxidative stress after pediatric TBI (Cousar et al., 2006). In contrast, HO-2 is constitutively expressed in neural tissues (Geddes et al., 1996). While it was proved that HO-2 can prevent cellular injury after TBI via inhibition of oxidative stress (Chang et al., 2003). Despite this pathophysiological understanding, the role of CO remains poorly understood in the process of HO metabolism.

The measurement of CO fluctuation and cellular distribution after TBI has been recently studied. Accumulating evidence shows that HO can successfully increase CO production and response to cellular stress (Carratu et al., 2003; Chang et al., 2003; Kanu et al., 2006). CO production rapidly increases in the brain following induction of various pathophysiological conditions in the brain, including acute hypotension, hypoxia, glutamate metabolism, and glutamatergic seizures (Parfenova and Leffler, 2008). Additionally, CO was found to have different therapeutic functions in different brain pathologies.

**FIGURE 2:**
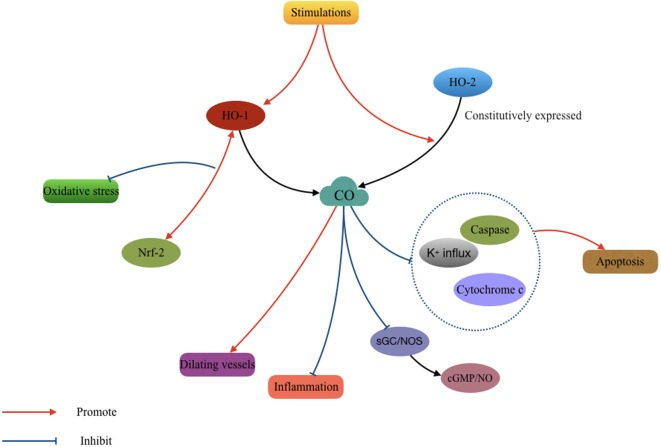
Mechanisms involved in the cytoprotective effect of carbon monoxide (CO) after TBI. The CO produced by heme oxygenase (HO) isozymes participated in the process of antioxidantien, anti-inflammation, anti-apoptosis, and vasodilatation.

As with NO, a very low concentration of CO can function as a vasodilator and a neurotransmitter in the brain (Zakhary et al., 1996; Leffler et al., 2006). Low concentrations of inhaled CO can prevent cerebral hypoxia and ischemia in occlusive cerebrovascular disease (Wang et al., 2011; Cai et al., 2017). Carbon monoxide-releasing molecules (CORMs)-A1 can reduce the inflammatory reaction in neuronal degenerative diseases (Chora et al., 2007). While in a mouse model of TBI, a recent study found that treatment with CORM-3 prevented the death of pericytes, thereby rescuing neural stem cells and ameliorating neurological impairment (Choi et al., 2016). The protective effect of CO appears to be related to the activation of sGC and NOS, namely cGMP and NO. However, a detailed mechanism was not described in these studies (Vieira et al., 2008; Queiroga et al., 2012; Schallner et al., 2013). In addition, CO inhibits oxidative apoptosis in the early phase following TBI by suppressing potassium influx, caspases activation, and cytochrome c release (Dallas et al., 2011). CO can also increase the interaction between Nrf2 and HO-1, effectively promoting HO-1 expression and increased antioxidant responses (Wang et al., 2011; **Figure 2**).

In conclusion, the mechanisms of vasodilation, anti-inflammation, anti-apoptosis, anti-proliferation, and anti-oxidant effects of CO need to be further investigated in the TBI model as they are potential targets for therapeutic intervention in TBI. Inhaled CO was rarely applied in past studies since the inhaled form is not tissue specific and the unnecessary CO can bring partial systemic hypoxia and toxicity. These complications make CORMs potential donors of CO (Queiroga et al., 2015). Meanwhile, CO administration should be further investigated in the future pre-clinical or clinical studies.

## Hydrogen Sulfide

Hydrogen Sulfide is another toxic gas that has important functions in physiological signal transduction (Liu H. et al., 2016). It can easily cross the cell membrane and enter intracellular compartments due to its high solubility in lipophilic solvents (Reiffenstein et al., 1992; Wang, 2002). H_2_S is produced from the cysteine degradation process by two pyridoxal-5^′^-phosphate (PLP)-dependent enzymes, namely cystathionine β-synthase (CBS) and cystathionine γ-lyase (CSE). CBS is expressed primarily in the nervous system, liver and kidney. While CSE is expressed in the cardiovascular system and liver (Lowicka and Beltowski, 2007). In addition, brain H_2_S was also found to be generated from cysteine with the assistance of 3-mercaptopyruvate sulfur transferase and cysteine amino transferase (Shibuya et al., 2009). It is involved in various biological functions after TBI including cerebrovascular regulation, oxidative stress reactions, inflammation, glutamate-mediated excitotoxicity, and apoptosis (Wang et al., 2014).

Recent studies demonstrated that the CBS and H_2_S levels in the brain were decreased during the early phase (12–24 h) and increased in the late phase (3–7 days) after TBI (Jiang et al., 2013; Zhang et al., 2013). These changes were closely related to levels of oxidative stress and the pathogenesis of TBI (Scheff et al., 2013). CBS activity was found to be up-regulated via the Calcium/calmodulin pathway and enhanced H_2_S production was found in response to glutamate (Eto and Kimura, 2002). However, CSE activity was less reported in the literature.

Using Sodium hydrosulfide (NaHS) as the H_2_S source, a study found a significant difference between TBI and NaHS-treated TBI mice in measures of neuronal morphology and the density of the hippocampus (Zhang et al., 2013). 90 or 180 μmol/kg of NaHS treatment can significantly reduce loss of the brain tissue and protect against the neuron damage. It suggested that H_2_S is also a neuroprotective gas for TBI treatment (Zhang et al., 2013). In addition, another study demonstrated that low dose NaHS (3 mg/kg) decreased the elevated BBB permeability, brain edema, and lesion volume in rats post-TBI. These effects were related to the activation of mitoK*_ATP_* channels and reduction of oxidative stress. However, a higher dose of NaHS (10 mg/kg) gave a worse outcome in this study, which draws attention to the importance of dosage of H_2_S supplement (Jiang et al., 2013). Furthermore, H_2_S was proved able to exert neuroprotection via inhibiting microglia activation following inflammatory effects and counteracts neurotoxicity. iNOS, NF-B, ERK, and p38 MAPK signaling pathways were inhibited in this process (Zhang Q. et al., 2014). Systemic administration of H_2_S has also been shown to significantly reduce brain edema and behavioral symptoms by anti-apoptosis and anti-autophagy effects. H_2_S reversed TBI-induced caspase-3 cleavage and Bcl-2 decline and prevented an increase in the Beclin-1/Bcl-2 ratio (Zhang M. et al., 2014; Fang et al., 2017).

**FIGURE 3:**
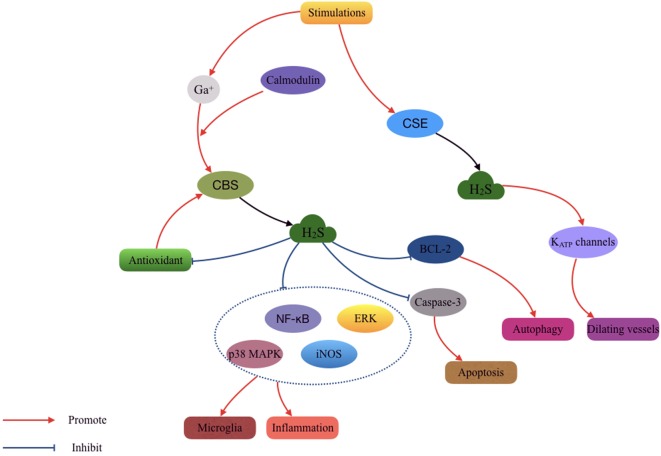
Mechanisms involved in the cytoprotective effect of hydrogen sulfide (H_2_S) after TBI. The H_2_S produced by cystathionine γ-lyase (CSE) and cystathionine β-synthase (CBS) isozymes participated in the process of antioxidantien, anti-inflammation, anti-apoptosis, anti-autophagy, and vasodilatation.

In addition, low concentrations of H_2_S may dilate cerebral vessels and protect against ischemia and hypoxia in the brain (Qu et al., 2006; Li et al., 2011). This effect is more likely led by CSE activation rather than CBS (Leffler et al., 2011). H_2_S activates K_ATP_ channels containing SUR2 subunits and acts on smooth muscle cells to promote vasodilation and subsequently maintain the CBF (Liang et al., 2011). However, fewer studies have investigated this effect in TBI models. The current understanding of the molecular mechanisms and biological roles of endogenous and exogenous H_2_S remains poor. Also, the study investigating the role of H_2_S-producing enzyme systems in TBI is unclear. This may be an area of focus in future H_2_S studies after TBI. Additionally, the use of NaHS and H_2_S in the clinical application requires optimal and safe concentration recommendations and strategy. Even a very low concentration (50 ppm) of inhaled H_2_S could lead to intense damage due to its high solubility. Thus, direct inhalation of H_2_S is not available (Qu et al., 2008; **Figure 3**).

## Hydrogen

Hydrogen (H_2)_ provides potential protective roles in neural diseases such as ischemic or hemorrhagic stroke (Cai et al., 2008; Zhan et al., 2012), TBI (Ji et al., 2010), CO poisoning (Sun et al., 2011), and neurodegenerative diseases (Fu et al., 2009). The underlying mechanisms may involve anti-oxygenation, anti-inflammation, anti-apoptosis effects, and BBB protection (Deng et al., 2014; Liu C.L. et al., 2016). The solubility of H_2_ is low in the normal environment, and no/few endogenous cells produce H_2_ in the mammalian CNS (Levitt, 1969; Sahakian et al., 2010). The therapeutic use of exogenous H_2_ in neural diseases is under investigation. The main donor of exogenous H_2_ in past research includes intravenous fluid of hydrogen-rich saline (Ono et al., 2011), potable H_2_ water (Ishibashi et al., 2012), and inhaled H_2_ gas (Nakao et al., 2010).

Inhalation of 2% H_2_ from 5 min to 5 h after TBI was shown to attenuate BBB damage, brain edema, lesion volume, and improved neurological outcome. The potential mechanism might be associated with decreasing oxidative products (8-iso-PGF2α and MDA) and promotion of endogenous antioxidant enzymatic activity (SOD and CAT) (Ji et al., 2010). Similarly, another study found that 2.9% H_2_ inhalation showed similar effects in brain tissues after surgery. However, this treatment failed to present the anti-oxidative or anti-inflammatory effects (Eckermann et al., 2011). In addition, hydrogen-rich saline facilitated synaptic plasticity and improved cognition after mild TBI. The hydrogen-rich saline protected TBI rat model through inhibition of oxidative damage and maintaining energy homeostasis (Hou et al., 2012). Recently, molecular hydrogen given in drinking water (mHW) was shown to relieve the acute alterations and neurodegenerative changes after TBI in a controlled cortical impact (CCI) model. The mHW alleviated brain edema, BBB disruption, and maintained normal brain interstitial fluid circulation. In addition, mHW increased ATP and nucleotide binding after TBI and inhibited pathological gene expressions that regulate oxidation/carbohydrate metabolism and suppressed cytokine activation (Dohi et al., 2014). In another study, pro-inflammatory cytokines (TNF-α, IL-1β, and HMGB1), inflammatory cell numbers (Iba1), and inflammatory metabolites (Cho) were attenuated, and anti-inflammatory cytokine (IL-10) was elevated after hydrogen-rich water therapy (Tian et al., 2016). In addition, H_2_-rich water can also up-regulate the expression of Nrf2 which prevents oxidative damage in TBI-challenged rats (Yuan et al., 2015).

**FIGURE 4:**
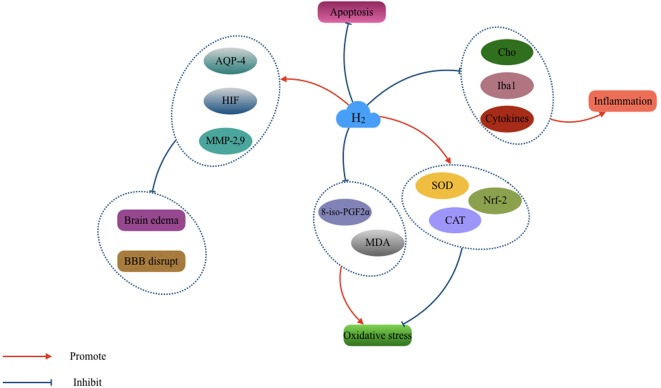
The cytoprotective effect of H_2_ in the pathological changing after TBI. hydrogen (H_2_) acts essential role in the antioxidant, anti-inflammation, anti-apoptosis, and vasodilatation. It also can relieve the brain edema and blood–brain barrier (BBB) disruption after TBI.

Based on the neuroprotective effects of H_2_ published in the past, H_2_ could be a promising therapy for clinical application (**Figure 4**). However, the adverse effects have not been well investigated. Researchers reported that some biological enzymes would decline upon ingestion of a certain concentration of H_2_which may bring a potential toxicity. The intervention strategies and concentrations of H_2_ used also differed among previous studies. Future studies should also focus on interactions between the anti-oxygenation, anti-inflammation, and anti-apoptosis effects induced by H_2_ therapy.

## Conclusion and Prospects

Biological gasses have smaller molecular weights compared to chemically formulated drugs. Thus, they can easily cross the BBB and diffuse to the brain tissues. Increasing evidence has demonstrated the potential clinical value of neuroprotective gasses in the treatment of neural diseases, including TBI. Endogenous gaseous are up-regulated during the pathological changes occurring after TBI, including redox reactions, inflammation, apoptosis, and excitotoxicity. Understanding the roles of endogenous gaseous molecules in different stages after TBI and determining an appropriate application strategy for exogenous gaseous molecules might provide us with more treatment options and significantly improve post TBI symptoms and outcome. However, the interaction between gasses and pathology is not well understood, and the application paradigms differ among published studies. The administration differences include in the gas source and gas ingestion methods, as well as the ideal concentrations needed for optimal results. In addition, safety and toxicity remain to be fully understood. Studies in this topic of TBI treatment could also focus on complicated aspects not studied or not elucidated in the current literature to help with the transition from current pre-clinical studies into future clinical studies.

## Author Contributions

SC was the principal investigator. XC and YF wrote the paper and made the original figures. XS and JW revised the figures. XH and CR handled the language and made some comments.

## Conflict of Interest Statement

The authors declare that the research was conducted in the absence of any commercial or financial relationships that could be construed as a potential conflict of interest.
